# High Functioning Autism with Missense Mutations in Synaptotagmin-Like Protein 4 (SYTL4) and Transmembrane Protein 187 (TMEM187) Genes: SYTL4- Protein Modeling, Protein-Protein Interaction, Expression Profiling and MicroRNA Studies

**DOI:** 10.3390/ijms20133358

**Published:** 2019-07-09

**Authors:** Syed K. Rafi, Alberto Fernández-Jaén, Sara Álvarez, Owen W. Nadeau, Merlin G. Butler

**Affiliations:** 1 Departments of Psychiatry & Behavioral Sciences and Pediatrics, University of Kansas Medical Center, Kansas City, KS 66160, USA; 2 Department of Pediatric Neurology, Hospital Universitario Quirón, 28223 Madrid, Spain; 3 Genomics and Medicine, NIM Genetics, 28108 Madrid, Spain; 4 Department of Biochemistry and Molecular Biology, University of Kansas Medical Center, Kansas City, KS 66160, USA

**Keywords:** autism candidate genes, synaptotagmin-like protein 4 (*SYTL4*), transmembrane protein 187 *(TMEM187*), SYTL4-protein structure, STRING-protein-protein interaction, expression profile, microRNA- interactions

## Abstract

We describe a 7-year-old male with high functioning autism spectrum disorder (ASD) and maternally-inherited rare missense variant of Synaptotagmin-like protein 4 (*SYTL4)* gene (Xq22.1; c.835C>T; p.Arg279Cys) and an unknown missense variant of Transmembrane protein 187 (*TMEM187*) gene (Xq28; c.708G>T; p. Gln236His). Multiple in-silico predictions described in our study indicate a potentially damaging status for both X-linked genes. Analysis of predicted atomic threading models of the mutant and the native SYTL4 proteins suggest a potential structural change induced by the R279C variant which eliminates the stabilizing Arg279-Asp60 salt bridge in the N-terminal half of the SYTL4, affecting the functionality of the protein’s critical RAB-Binding Domain. In the European (Non-Finnish) population, the allele frequency for this variant is 0.00042. The *SYTL4* gene is known to directly interact with several members of the RAB family of genes, such as, *RAB27A, RAB27B, RAB8A,* and *RAB3A* which are known autism spectrum disorder genes. The *SYTL4* gene also directly interacts with three known autism genes: *STX1A*, *SNAP25* and *STXBP1.* Through a literature-based analytical approach, we identified three of five (60%) autism-associated serum microRNAs (miRs) with high predictive power among the total of 298 mouse Sytl4 associated/predicted microRNA interactions. Five of 13 (38%) miRs were differentially expressed in serum from ASD individuals which were predicted to interact with the mouse equivalent *Sytl*4 gene. *TMEM187* gene, like *SYTL4*, is a protein-coding gene that belongs to a group of genes which host microRNA genes in their introns or exons. The novel Q236H amino acid variant in the TMEM187 in our patient is near the terminal end region of the protein which is represented by multiple sequence alignments and hidden Markov models, preventing comparative structural analysis of the variant harboring region. Like *SYTL4*, the *TMEM187* gene is expressed in the brain and interacts with four known ASD genes, namely, *HCFC1; TMLHE; MECP2*; and *GPHN. TMM187* is in linkage with *MECP2*, which is a well-known determinant of brain structure and size and is a well-known autism gene. Other members of the *TMEM* gene family, *TMEM132E* and *TMEM132D* genes are associated with bipolar and panic disorders, respectively, while *TMEM231* is a known syndromic autism gene. Together, *TMEM187* and *SYTL4* genes directly interact with recognized important ASD genes, and their mRNAs are found in extracellular vesicles in the nervous system and stimulate target cells to translate into active protein. Our evidence shows that both these genes should be considered as candidate genes for autism. Additional biological testing is warranted to further determine the pathogenicity of these gene variants in the causation of autism.

## 1. Introduction

Whole exome sequencing (WES) and occasionally whole-genome sequencing (WGS) are increasingly used in clinical practice for diagnosis, medical intervention and prognosis [1,2,3]. High heritability estimates and family studies have supported a definite role of genetics in autism spectrum disorder (ASD). In neurodevelopmental disorders, particularly ASD and intellectual disability have diagnostic rates using WES which fluctuates in part from differences in clinical features from one study to another with rates up to 50% [4,5,6,7]. We describe a 7-year-old male with high-functioning autism spectrum disorder presenting for genetic services and whole exome sequencing following a normal microarray analysis, and variants were found in two X-linked genes: *SYTL4* and *TMEM187*.

### 1.1. Synaptotagmin-Like Protein 4 (SYTL4) Gene

**Figure 1:**
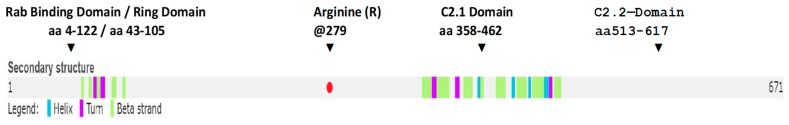
Graphic representation of the secondary structure of *SYTL4* gene indicating the location of Rab binding domain, ring domain, C2 domains, and the location of arginine at amino acid position 279.

**Figure 2:**
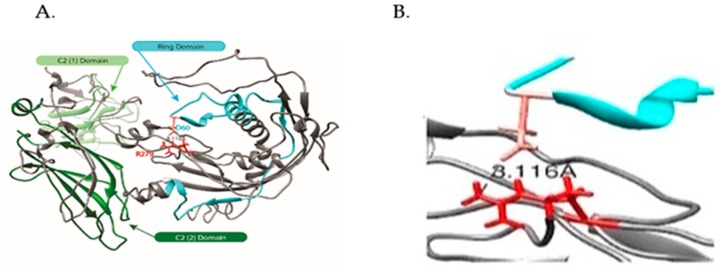
The native SYTL4 protein structure contains the C2 domains, ring domain, and an apparent salt bridge between arginine (R279) and aspartic acid (D60) as noted in (**A**). Arginine 279 (side chain in red) is part of a large extended loop conformation that appears to be stabilized by an apparent salt bridge formed between it and Asp60 (side chain in Salmon) in the ring domain. The distance (3.116Å) calculated between the Arg guanidinium nitrogen and Asp carboxyl oxygen is well within the threshold distance observed for salt bridges in a comprehensive survey of crystal structures as noted in (**B**).

*SYTL4* gene, also known as Granuphilin/SLP4, encodes a member of the synaptotagmin-like protein family. Members of this family are characterized by an N-terminal RAB27-binding domain and C-terminal tandem C2 domains (Figure 1 and Figure 2). The first C2 domain binds phospholipids in a calcium-independent manner, whereas the second C2 domain does not (http://omim.org/entry/300723). The encoded protein binds to specific small RAB-GTPases involved in intracellular membrane trafficking. This protein binds to RAB27 and may be involved in inhibiting dense core vesicle exocytosis. Alternate splicing results in multiple transcript variants that encode the same protein.

*SYTL4* gene is relevant in neuronal system development and implicated in neurological and psychological diseases [8] (Entrez Gene ID # 94121 (Human); ID # 27359 (Mouse). This gene is highly expressed in the bed nucleus of the stria terminalis (BNTS) [9], a brain region that regulates mood, motivation for social behavior and social attachment [10]. *SYTL4* expression is down regulated in the dorsal raphe nucleus from patients with major depressive disorder [11]. In a mouse model of anxiety, significant change in Sytl4 was observed among the altered protein networks in the brain proteome [12].

Targeted Null/Knockout mutant *Sytl4* mammalian phenotypes for *Sytl4* showed abnormal behavior and neurological phenotype [13,14,15]. This gene interacts directly with other known autism genes. Confirmatively, gene expression knock-out *Sytl4* mutant mouse includes abnormal behavior and neurological problems [13,14]. Diseases already associated with *SYTL4* include Epileptic Encephalopathy (early Infantile) and Branchiootic syndrome (www.genecards.org/cgi-Fbin/carddisp.pl?gene= SYTL4&keywords=sytl4). A missense mutation in *SYTL4* was also recently reported in a female with autism and non-skewed X-chromosome inactivation [16].

### 1.2. Transmembrane Protein 187 (TEM187) Gene

The second gene in our study, *TMEM187*, is additionally known as *ITBA1/CXORF12/DXS9878E*- gene and consists of two exons which encode a multi-pass membrane protein expressed in all regions of the brain (www.genecards.org/cgi-bin/carddisp.pl?gene=TMEM187&keywords=TMEM187). This is a conserved gene that codes a 261-amino-acid protein with six transmembrane helical domains. Unlike *SYTL4*, not much is known about this gene, but it belongs to a group of genes which host microRNA genes in their introns or exons [17]. One of this gene’s related phenotypes is schizophrenia (GWAS catalog for *TMEM187* gene: Gene relation via enhancers containing phenotype SNP: Enhancer ID: GH0XJ153980). The latest STRING- network of protein interactions for this gene reveals that it directly interacts with four known ASD genes, namely, *HCFC1; TMLHE; MECP2* and *GPHN*. (https://gene.sfari.org/database/human-gene/).

## 2. Results

### 2.1. Genomic Study

Whole exome sequencing performed in trios revealed maternally inherited X-linked missense variants: c.835C>T, p. Arg279Cys in the *SYTL4* gene and c.708G>T; and p. Gln236His in the *TMEM187* gene. Research findings will be discussed that relate specifically to *SYTL4* and *TMEM187* gene variants seen in our patient and as emerging candidate genes for autism.

### 2.2. Synaptotagmin-Like 4 (SYTL4) Gene

#### 2.2.1. Deleterious and Damaging Nature of the *SYTL4*- Variant

The maternally inherited variant in Synaptotagmin-like protein 4 gene *(SYTL4*) at chromosome-X position g.99944930G>A, c.835C>T, p. Arg279Cys [NM_001174068.1] sits within exon 9 of the *SYTL4* gene located at the Xq22.1 cytoband (Figure 1). This particular *SYTL4* nucleotide variant CGC⇒TGC at position 835 (exon 9; alternating) has been predicted by large-scale genomic sequencing studies, such as the 1000 Genome project, to be deleterious (SIFT score 0.01) and possibly damaging (Polyphen score 0.79), which is further validated with a MAF score of 0.0005/2. The Exome Aggregation Consortium (ExAC) database including data from 1000 genomes further indicated that the *SYTL4* variant is also damaging.

Our patient is from Spain and according to Exome Aggregation Consortium, the allele frequency among European (Non-Finnish) population for this variant is 0.0004222. No homozygotes were found, but eight hemizygotes (male) were observed in 47372 alleles which presumably included both isoforms. There is no reported information from these individuals regarding findings related to ASD when examining clinical information-sharing resources such as Phenome Central, Gene Matcher or ClinVar.

#### 2.2.2. Modeling of Native and R279C Mutant for *SYTL4* Gene

There is limited high-resolution structural information about *SYTL4*. The crystal [18] and NMR [19] structure of C2(1) (residues 354-483) and ring domain (residues 43-105) cover only 29% of the protein primary structure. A structural comparison of *SYTL4* and the R279C mutant was carried out using the I-TASSER multiple threading approach [20]. The protein structure closest to both forms of SYTL4 found in the Protein Data Bank (PDB) [21] is human synaptotagmin 2 (PDB #4P42) [22,23], with TM scores (0.658 and 0.661) and RMSD values (1.15 Å and 0.96 Å), respectively, for the native and R279C form (Figure 2).

#### 2.2.3. Hierarchical Protein Structural Modeling Study of Both Native and R (279) C SYTL4

**Figure 3:**
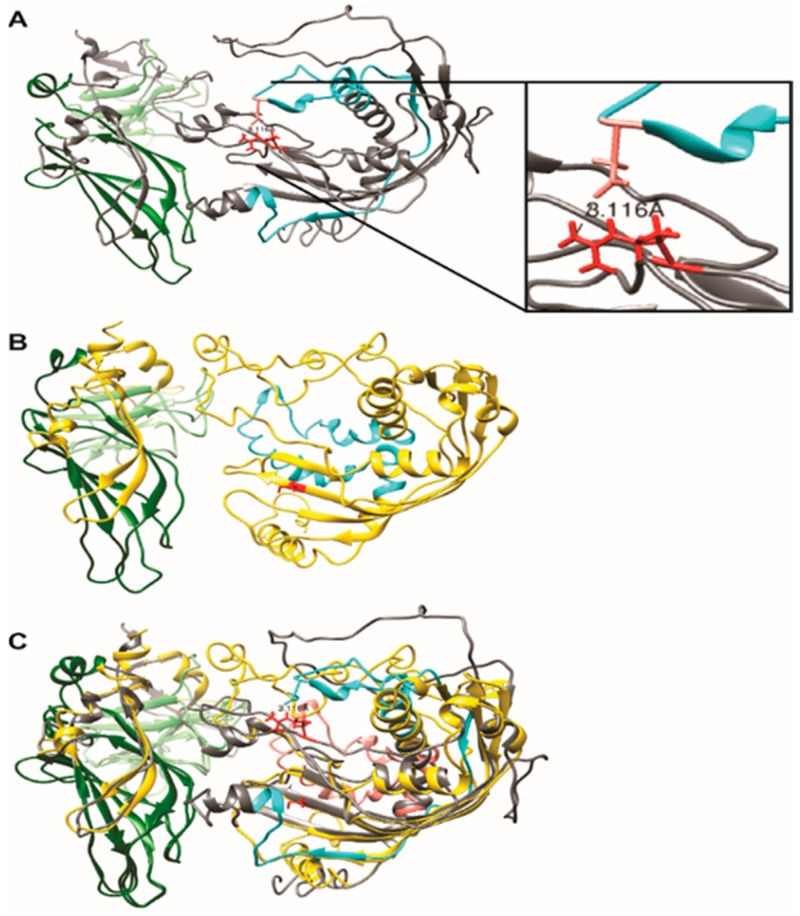
Effect of the R[Arg]⇒C[Cys] amino acid change at 279 on the structure of SYTL4 Proteins. Theoretical 3D structures of native and R279C SYTL4. Hierarchical protein structural modeling of both native (**A**) and R279C (**B**) SYTL4 proteins were carried out using I-TASSER. Three X-ray crystal structures of human Synaptotagmin C2 domains (Protein Data Bank ID: 2R83, 4P42 and 3HN8) among the top 10 templates used for both SYTL4 forms. Native SYTL4 (grey ribbon trace) and R279C variant (gold ribbon trace) are shown with C2(1), C2(2) and ring domains rendered as light green, dark green and cyan traces, respectively, in Panels (**A**,**B**). Panel (**A**): SYTL4 native protein structure indicating the C2 domains, ring domain, and an apparent salt bridge between arginine (R279) and aspartic acid (D60). Shown magnified in the native SYTL4 structure is the apparent salt bridge formed between D60 and R279. Panel (**B**): SYTL4 R279C variant (gold ribbon trace) shows significant displacement of both the 279 amino acid site (salmon ribbon trace) and ring domains of native (cyan ribbon trace). Panel (**C**): An overlay of native **(A)** and R279C (**B**) SYTL4 structures demonstrates relatively good alignment for the C terminal C2 domains and confirms the significant displacement of both the 279 amino acid site (salmon ribbon trace) and ring domains of native (cyan ribbon trace) SYTL4.

Both atomic models preserved the canonical C2 structures of synaptotagmins; [21,22,23], however, only the native SYTL4 preserved the extended structure of the ring domain (Figure 3) [18]. An overlay (Figure 3C) of two atomic models demonstrated good alignment of the C-terminal C2 domains, whereas the structures of the region comprising both the ring domain and the 279 amino acid change sites for the two SYTL4 forms differed considerably. In the native SYTL4 model, Arg 279 (side chain in red) is part of a large extended loop conformation that appears to be stabilized by an apparent salt bridge formed between it and Asp60 (side chain in Salmon) in the ring domain. The distance (3.116 Å) calculated between the Arginine guanidinium nitrogen and Asp carboxyl oxygen (magnified in Figure 3B) is well within the threshold distance observed for salt bridges in a comprehensive survey of crystal structures [24]. In the R279C model, Cys279 is in a beta sheet, with no apparent hydrogen bonding contacts observed within distance constraints for such interactions [25] (Figure 3A,B). Our modeling suggests a potential structural change induced by the R279C variant, which eliminates the stabilizing Arg279-Asp60 salt bridge and, therefore, leads to significant structural changes in the N-terminal half of the SYTL4.

#### 2.2.4. STRING- Protein–Protein Interaction Network Study Reveals Direct Interaction of *SYTL4* with Other Known Autism Genes

**Figure 4:**
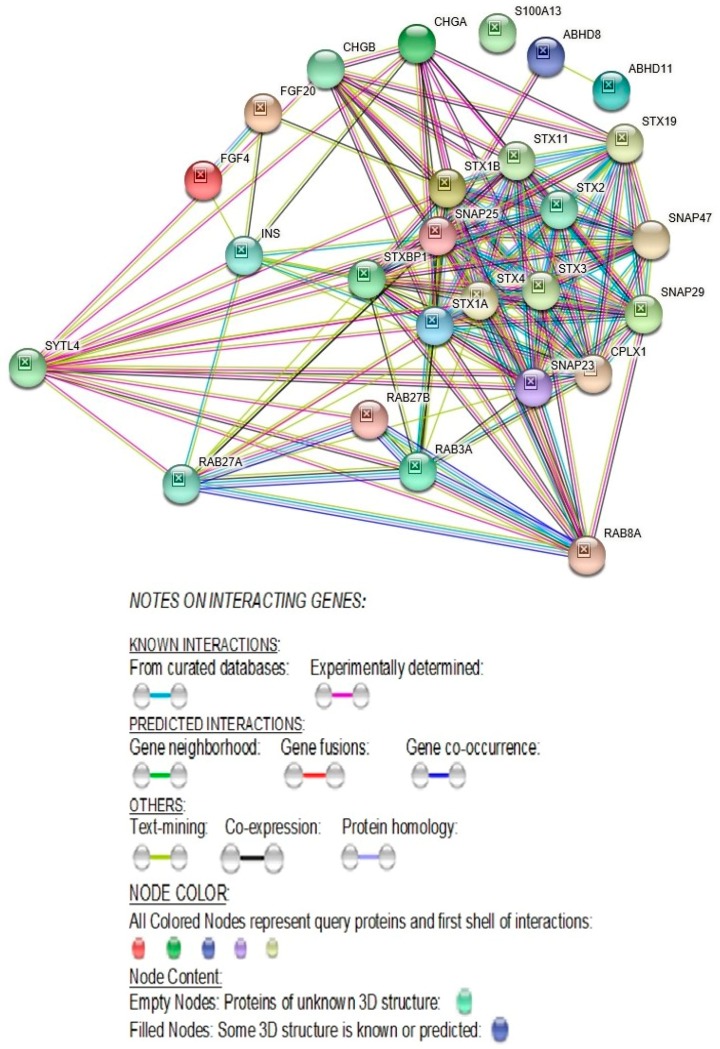
STRING- Protein-Protein Interaction Network of SYTL4. [https://version11.string-db.org/cgi/network.pl?taskId=f7upTuHlbV0A]. SYTL4- Interacting ASD Genes; *SYTL4*: Synaptotagmin-like protein 4; modulates exocytosis of dense-core granules and secretion of hormones in the pancreas and the pituitary. Interacts with vesicles containing negatively charged phospholipids in a Ca (2+)-independent manner; synaptotagmin-like tandem C2 proteins. STXBP1: Syntaxin-binding protein 1; may participate in the regulation of synaptic vesicle docking and fusion. Essential for neurotransmission and binds syntaxin, a component of the synaptic vesicle fusion machinery (Score: 0.746). STX1A: Syntaxin-1A; plays a role in hormone and neurotransmitter exocytosis. Potentially involved in docking of synaptic vesicles at presynaptic active zones (Score: 0.805). SNAP25: Synaptosomal-associated protein 25; t-SNARE involved in the molecular regulation of neurotransmitter release. May play an important role in the synaptic function of specific neuronal systems (Score: 0.551).

SYTL4 directly interacts with three known *ASD* genes, *STX1A, SNAP25* and *STXBP1* (https://gene.sfari.org/database/human-gene/). *SYTL4* also interacts with 14 other genes, namely, *FGF4*, *STX1B*, *SNAP29*, *RAB3A*, *SNAP23*, *RAB27B*, *RAB8A*, *SNAP47*, *STX4*, *STX19*, *STX3*, *STX11*, *STX2*, and *RAB27A*, whose transcriptome-wide isoform-level multiple related gene family members are known ASD genes (https://gene.sfari.org/database/human-gene/). SYTL4 also directly interacts with several members of the RAB-family of genes, such as *RAB27A*, *RAB27B*, *RAB8A*, and *RAB3A* [26] (Figure 4).

#### 2.2.5. SYTL4- Molecular Pathways and Associated Diseases

The molecular pathways of the *SYTL4* gene includes a synaptic vesicle cycle, insulin secretion, AMPK- signaling, and SNARE interactions in vesicular transport (Table 1). Diseases associated with synaptic vesicle cycle defects include early infantile epileptic encephalopathy and defects in insulin secretion including defects in degradation of gangliosides which are abundantly expressed in the nervous system. The deficiency of certain gangliosides will affect the regenerative ability of injured hypoglossal nerves [27]. Lastly, defective SNARE interactions in vesicular transport have been implicated in CEDNIK-syndrome, which includes cerebral dysgenesis and neuropathy. All the noted pathways and their associated diseases broadly impact nervous system function which are relevant to autism (Table 1) [26].

#### 2.2.6. SYTL4- Networks of Biological Processes

As presented in Table 2 [26], extensive biological processes of the *SYTL4* gene pertain to synaptic vesicle functions, including neurotransmitter secretion and regulation of signaling, RAB protein signal transduction, glutamate secretion, neuro-muscular synaptic transmission, and axonogenesis, all of which are relevant for proper neuronal function, and thus important in autism.

#### 2.2.7. SYTL4- Molecular Functions

The molecular function of the *SYTL4* gene includes syntaxin binding, which is essential for neurotransmission (Table 3) [26] and directly interacts with syntaxin-binding protein 1 (STXBP1) (Figure 4). Mutations in STXBP1 are associated with infantile-epileptic encephalopathy-4 [28]. *SYTL4* molecular gene function also relates to synaptosomal-associated protein receptor (SNAPRe) activity which regulates neurotransmitter release to ensure vesicle-to-target specificity [29]. In addition, the molecular function of the *SYTL4* gene pertains to trimeric-G-protein (GDP binding protein) which plays a pivotal role in signal transduction pathways for numerous hormones and neurotransmitters [30].

#### 2.2.8. Missense Mutation Causing R (279) C Amino Acid Change Affects the Structures of Canonical *SYTL4* Gene as Well as Its Shorter Isoform

**Figure 5:**
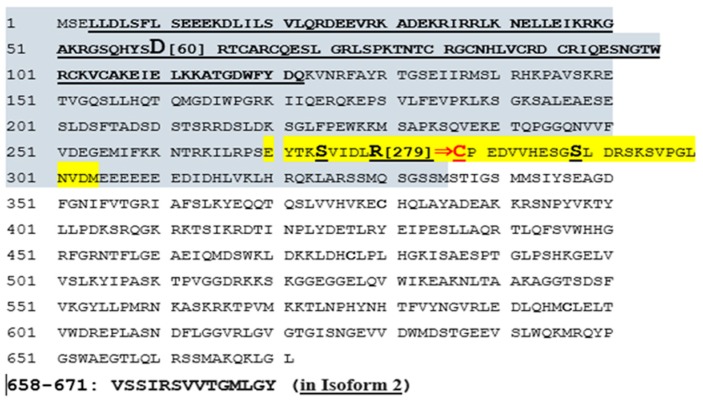
SYTL4 amino acids sequence data for the “canonical” form and its truncated isoform 2, both encompassing the alternating exon 9 with the missense mutation resulting in R[Arg]⇒C[Cys] at 279: Amino acid sequences that are highlighted in blue are the first 335 amino acids which are common to both the full length ‘canonical’ sequence of the SYTL4 protein and its truncated isoform 2, both containing the alternating exon 9 Glu(E) 270–Met(M) 304, highlighted in yellow with the R[Arg]⇒C[Cys] at 279. The location of native arginine residue (at 279) in-between two Serine(S) amino acid residues at 274 and 289 positions (which have been shown to undergo post-translational phosphorylation) is underlined. The RAB-Binding Domain (comprised of amino acids 4 through 122) is also indicated with underline, within which lies D[Asp] at position 60 and takes part in the apparent salt bridge formation with R[Arg] at 279 in the native protein configuration. The presence of mutant C[Cys] at 279 leads to the formation of an extended beta-pleated sheet therein instead (as seen in Figure 3A,B).

The amino acid change found in our study, R(arg)279C(cys) in exon 9 (Figure 1), is integral for the full length ‘canonical’ sequence of the SYTL4 protein with 671 amino acids. It is also part of the shorter isoform containing 349 amino acids shown in Figure 5. The R>C amino acid variation at residue 279 has been classified by the Human Genome Variation Society (HGVS) as a missense variant (variation ID # rs141441277) and determined to be least common with a Minor Allele Frequency (MAF) of 0.000529801. It is further predicted as “possible damaging” with a PolyPhen score of 0.79, while SIFT predicts the effect of this amino acid change on protein function as “deleterious” (https://gnomad.broadinstitute.org/variant/X-99944930-G-A; Variant Effect Predictor (VEP) program: https://useast.ensembl.org/info/docs/tools/vep/index.html.

#### 2.2.9. Autism Predictive Human Serum MicroRNAs with Predicted Interaction with Mouse *Sytl4* Gene

Among the total of 298 mouse Sytl4 associated/predicted miRs, three of the five miRs (60%), namely, miR181b-5p, miR320a, and miR130a-3p- are with good ASD predictive power in serum, as identified in Table 4. Apart from the five-serum miRNAs with good ASD predictive power, miR106b-5p and miR328- were up and down regulated (respectively) in the serum ASD study. Both miRs have been reported as showing altered expression among schizophrenics [31,32,33,34]. Interestingly, these two miRs have been predicted to interact with mouse *Sytl4* gene (Table 4). In addition, four other miRs (miR98, miR103, miR132, and miR320) have been found to be dysregulated in the superior temporal gyrus of ASD [35] and predicted to interact with mouse *Sytl4* gene. Furthermore, miR106b, miR181b-5p, miR320, and miR328 are differentially expressed in the ASD cerebellar cortex [35,36] and predicted to interact with mouse *Sytl4* gene (Table 4).

### 2.3. Transmembrane Protein 187 (TEM187) Gene

#### 2.3.1. Structure of *TMEM187* Gene, Expression and Location of the Novel Variant

**Figure 6:**
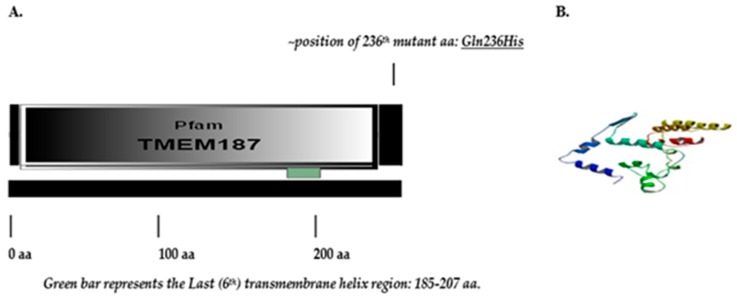
(**A**). Graphic representation of TMEM187 protein indicating the location of Q236H amino acid variation beyond the last (6th) transmembrane helical domain (191-210aa), at the near terminal end of the *Pfam* domain (8-245aa) (https://pfam.xfam.org/family/tmem187; TMEM187 (PF15100)). (**B**). Theoretical partial 3D protein structure of the encoded multi-pass transmembrane protein targeting the 15-200aa region (https://modbase.compbio.ucsf.edu/), excluding the Q236H amino acid variant near the terminal end region of the protein which is represented by multiple sequence alignments and hidden Markov models preventing comparative structural analysis of the variant harboring region.

*TMEM187* gene consists of two exons and encodes a multi-pass membrane protein (Figure 6).

The maternally inherited *TMEM187* missense variant lies beyond the last transmembrane helix region at the near terminal end of the *Pfam* domain 8-245aa represented by multiple sequence alignments and hidden Markov models (https://pfam.xfam.org/family/tmem187; TMEM187 (PF15100)). The *TMEM187* gene has three transcripts (splice variants) and 35 orthologs.

#### 2.3.2. Deleterious and Damaging Nature of the Novel *TMEM187* Gene Variant

Unlike the *SYTL4* gene variant, the *TMEM187* missense gene variant (c.708G>T; p. Gln236His) is neither found in the Exome Aggregation Consortium (ExAC) database (which includes the 1000 genome data) or in the *Ensemble.org* data base. The nature of this novel Glutamine(Q)236 to Histidine(H) variation in TMEM187 is considered deleterious as determined by the PROVEAN (Protein Variation Effect Analyzer v1.1) score of = −4.474 with prediction cutoff of −2.5; SIFT score as deleterious (0.05); and PolyPhen-2 score as probably damaging (0.432). (http://gnomad.broadinstitute.org/variant/X-153248221-G-T). PROVEAN prediction generated score of −4.47 is termed as deleterious (scores <−2.5 considered deleterious), while the SIFT score of 0.05 approaches 0.0 which is the most damaging score.

#### 2.3.3. TMEM187 Gene Is Expressed in the Brain

The *TMEM187* gene is ubiquitously expressed in all systems including all parts of the brain (www.uniprot.org/uniprot/Q14656; www.genecards.org/).

#### 2.3.4. Latest STRING- Gene Interaction Network Study Reveals Direct Protein–Protein Interactions of TMEM187 with Several Other Known Autism Genes

**Figure 7:**
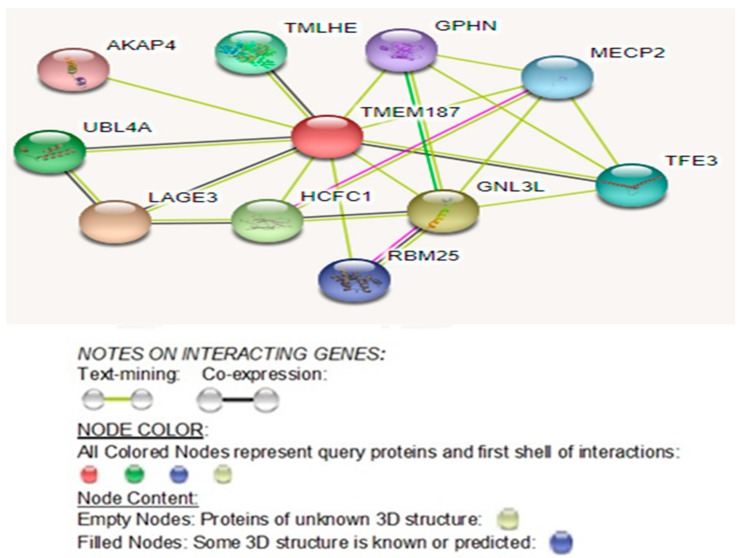
STRING-Protein–Protein Interactions of TMEM187. (https://string-db.org/network/9606.ENSP00000358999). TMEM187- Interacting ASD Genes: HCFC1: *Host cell factor 1*: Involved in control of the cell cycle; Coactivator for EGR2 and GABP2 (Score: 0.747); TMLHE: Trimethyllysine dioxygenase, mitochondrial: Converts trimethyllysine (TML) into Hydroxytrimethyllysine (HTML). (Score: 0.657); MECP2: Methyl-CpG-binding protein 2: Chromosomal protein that binds to methylated DNA; Mediates transcriptional repression through interaction with histone deacetylase and the corepressor SIN3A (Score: 0.555); GPHN: Gephyrin: Microtubule-associated protein involved in membrane protein-cytoskeleton interactions (Score: 0.531).

Analysis of its STRING- network of interactions (Figure 7) reveals that it directly interacts with four known ASD genes, namely *HCFC1*, *TMLHE*, *MECP2*, and *GPHN* (https://gene.sfari.org/database/human-gene/). Moreover, three other directly interacting genes, namely *UBL4A, RBM25*, and *AKAP4,* though not known ASD genes, do show other transcriptome-wide isoform-level family member genes known as ASD genes: *UBL7*, *RBM27*, *RBM8A*, *RBMS3*, and *AKAP9* (https://gene.sfari.org/database/human-gene/).

## 3. Discussion

Our research findings will be discussed related specifically to both the *SYTL4* and *TMEM187* gene variants seen in our patient and evidence for the two gene variants playing a role in the causation of high-functioning autism spectrum disorder.

### 3.1. Synaptotagmin-Like 4 (SYTL4) Gene

As an emerging candidate gene for autism with analyzed protein modeling, protein interactome networks, expression profiling and microRNA interactions will follow.

#### 3.1.1. Protein Structure Altering Rare Variants Have Been Observed to Be More Frequent in Individuals with Autism

Though common variants are a large driving factors in autism spectrum disorder, the effect size of individual common variants is estimated to be small [38,39]. Therefore, the search for rare variants exhibiting a much larger individual effect is ongoing. Protein structure-altering rare variants have been observed more frequently in ASD cases, and ASD risks are increased when two rare variants may deleteriously affect both copies for an autosomal protein, or a single copy of an X-chromosomal protein among ASD males [40]. Rare hemizygous mutations on the X-chromosome are found to be more enriched in male ASD patients compared to controls. Furthermore, if rare hemizygous mutations on the X-chromosome in males alter gene expression known to be present in the brain, then the overall odds-ratio for ASD will be increased [41].

#### 3.1.2. Deleterious and Damaging Nature of the *SYTL4* Gene Variant

It is important to note that this particular SYTL4 nucleotide variant CGC⇒TGC resulting in p. Arg279Cys (Figure 1) has been predicted through large-scale genomic sequencing studies, such as the 1000 Genome project, and has been judged to be deleterious (SIFT score 0.01) and possibly damaging (Polyphen score 0.79) with a MAF score of 0.0005/2.

It should also be noted that ExAC database which includes the 1000 genomes data, also calls this SYTL4 variant damaging and deleterious based on Polyphen and SIFT, respectively. Our patient being from Spain, according to ExAC, among the European (Non-Finnish) population, the allele frequency for this variant is 0.0004222 without any homozygotes and with eight hemizygotes (male) for 47,372 alleles, which presumably includes both isoforms. There is no information available as to any of these eight hemizygotes, or any other hemizygote being reported as exhibiting ASD in any of the clinical information-sharing resources such as Phenome Central, Gene Matcher and ClinVar.

#### 3.1.3. Randomness of X Chromosome Inactivation Could Render the Mother Asymptomatic

It is important to note that our male heterozygous carrier of the R (279) C variant is expected to be fully penetrant for the mutant allele. The proband’s mother is a heterozygous carrier of the R (279) C variant and due to randomness of X chromosome inactivation may render her as asymptomatic given that the X-linked *SYTL4* gene is not over expressed among females, indicating that it does not escape inactivation [42]. The proband’s mother is expected to express equally her normal SYTL4 allele along with the mutant allele.

#### 3.1.4. Our Modeling Results Show Large Conformational Changes Proximal to the R (279) C Amino Acid Variation

It is possible that the structure and function of both the canonical and the truncated isoform of SYTL4 protein will be affected, particularly considering their interactions at membrane surfaces. Our modeling results show large conformational changes proximal to the R (279) C amino acid variation within exon 9. The flanking regions contain two known phosphorylatable serines (YTKS (@274) VIDLR (@279) P EDVVHESGS (@289) L) as shown in Figure 5.

#### 3.1.5. Missense Mutations Change the Size or Properties of Amino Acids Preventing the Function of Proteins

A study (reported by our co-authors: SKR, MGB) in whole exome sequencing in females with autism, a non-synonymous missense mutation (X: 99941091; C>G; p.H448D) of the SYTL4 gene was observed in a female with autism and random X-chromosome inactivation (46–54%). This patient additionally harbored four other autosomal missense gene mutations [16]. Missense mutations are of importance in understanding the structure or function of a protein since they usually occur in amino acid residues of structural or functional significance by changing the size or properties of the amino acid there by preventing the function of that protein [43,44]. The effect of such a mutation is additionally dependent on the sequence and structure context of the alteration [45]. Proteins fold according to minimum free energy [46]. Only correctly folded proteins can deliver the functional properties of a protein, and even minor changes in the size or properties of an amino acid side chain can alter or prevent the function of the protein [47]. On the other hand, even large deletions or insertions may be tolerated in numerous positions within a protein [48].

Protein function and interactions require both stability and specificity. Since most disease-causing mutations produce structural effects, the importance of a specific gene location and protein production is emphasized [47]. Structural information is needed to fully understand the effects and consequences of mutations, whether disease-causing or used purposefully to modify the properties of a protein. Three-dimensional structures and computer models have been used to elucidate disease mechanisms from specific amino acid substitutions [49,50]. For example, in a study of 4236 mutations from 436 genes, mutations at arginine and glycine residues are collectively responsible for about 30% of genetic diseases [43].

#### 3.1.6. SYTL4 Amino Acid Change R (279) C in Exon 9: RAB-Binding Domain

The amino acid change in our study patient, *SYTL4* R(279)C in exon 9 (Figure 1, Figure 3 and Figure 5), has been determined to severely affect the critical functioning of this gene’s encoded RAB protein binding region at its N-terminal [51,52], which is perhaps analogous to the effect of a significant change in Sytl4 gene expression observed among the altered protein networks in the mouse brain proteome in a mouse model of anxiety [12]. The SYTL4 RAB-Binding Domain within which lies D[Asp], at 60 takes part in the apparent salt bridge formation with R[Arg] at 279 only in the native protein configuration with the presence of mutant C[Cys] at 279 leading to the formation of an extended beta-pleated sheet (see Figure 3A,B).

#### 3.1.7. Effect of the R[Arg]⇒C[Cys] Amino Acid Change at 279 on the functionality of the RAB-Binding Domain

Our analysis of the mutant and native SYTL4 protein structure models shows that in the native protein, arginine (R279) forms an apparent salt bridge with aspartic acid (D60) (Figure 2 and Figure 3A). This arginine is part of a large extended loop conformation that appears to be stabilized by the apparent salt bridge formed between it and Asp60 within the RAB-Binding Domain [aa 4-122] and the Ring Domain [aa 43-105] (Figure 2 and Figure 3A).

In our R (279) C mutant protein structure model (Figure 3B), cysteine (C279) is located amidst a beta sheet with no apparent hydrogen bonding contacts observed within distance constraints for such interactions. Our modeling suggests a potential structural change induced by the R (279) C variation eliminating the stabilizing Arg279-Asp60 salt bridge and leads to significant structural changes in the N-terminal half of SYTL4 (Figure 3B,C). This change could very well affect the functionality of the RAB-Binding Domain, not only for the canonical-full-length SYTL4 protein (isoform-1), but also for the truncated SYTL4 protein: isoform-2 (Figure 5).

#### 3.1.8. Potentially Deleterious R (279) C Amino Acid Change “Likely” To Affect Its Neighboring Active Phosphorylation Sites

Given our modeling and profiling results, large conformational changes were seen proximal to the R279C amino acid variation within exon 9. Flanking regions do contain two known phosphorylatable serines (YTKS (@274) VIDLR (@279) P EDVVHESGS (@289) L) (Figure 5). The structure and function of both canonical and truncated isoforms of the SYTL4 protein may be affected, particularly considering their interactions at membrane surfaces.

#### 3.1.9. Role of Arginine (R279) in SYTL4 Protein Structure and Function

The missense mutation identified in our study causes a change in the amino acid at 279 from R [Arginine] to C [Cysteine] (Figure 1 and Figure 2B). Arginine is a large polar amino acid with sidechains that prefers to reside in an aqueous environment and is found more commonly on the surface of a protein. SYTL4 is a protein that anchors to the cell membrane and is exposed to both sides of the membrane by aqueous environments of the luminal and cytoplasmic sides. The C2 domain of SYTL4 protein facilitates binding of this protein to cell membranes and is often found in the active centers of proteins that bind phosphorylated substrates [53,54].

The arginine (R279) residue is in-fact located in between two Serine(S) residues at 274 and 289- positions (YTKS (@274) VIDLR (@279) PEDVVHESGS (@289) L) (Figure 5) and is shown to undergo post-translational phosphorylation [55,56,57]. Arginine is capable of “salt bridging” by forming non-bonded and hydrogen-bonded paired electrostatic interactions between acidic carboxyl groups and basic amino groups in single or adjacent protein chains [58]. One important role of “salt bridging” is connecting protein subunits or joining two secondary structures to form quaternary structures where they can connect as many as five secondary structure units [58].

#### 3.1.10. Arginine Disfavors Cysteine for Substitution

Given the above contrasting basic differences between arginine and cysteine in their properties and their role in protein structure and function, arginine disfavors cysteine for substitution, particularly in extracellular and membrane proteins, such as SYTL4. Since such substitution can be devastating to protein stability and function given the loss of arginine’s ability to create stabilizing hydrogen bonds, the substituted cysteine residue’s ability to alter the native three-dimensional conformation of the protein molecule is important [44]. Thus, the potential for a structural and functional change induced by the SYTL4 R279C amino acid change is consistent with the noted physiochemical differences between arginine and cysteine [44].

#### 3.1.11. Dysfunction of Evolutionarily Conserved RAB-Binding GTPases Play a Role in Autism and Neuronal Disorders

Among the transcriptome-wide RAB family of genes, *RAB2A*, *RAB11FIP5*, *RAB19*, *RAB39B*, and *RAB43* are also known ASD genes [59,60,61] (https://gene.sfari.org/database/human-gene/) and SYTL4 protein interacts with several other members of the RAB family of proteins including RAB3A, RAB8A, RAB27A, and RAB27B (Figure 4). Therefore, the defect in the RAB protein-binding region in the N-terminal half of the SYTL4 protein is due to the R (279) C amino acid variant and may be a causal factor for the high-functioning autism in our patient.

In addition, the *SYTL4* gene directly interacts with Syntax Binding Protein 1 (STXBP1). Both SYTL4 and STXBP1 are known to interact with RAB3A. STXBP1 interaction with RAB3A promotes RAB3A dissociation from the vesicle membrane (https://www.uniprot.org/uniprot/P61764). SYTL4 protein additionally interacts with several other members of the RAB family of proteins, including RAB3A, RAB8A, RAB27A, and RAB27B. Further, RAB-binding domain of SYTL4 serves as a preferred effector binding site for the GTP-bound form of the RAB27A protein that regulates the exocytosis of secretary granules [62,63,64]. RAB27A binds to the N-terminus SLP homology domains 1 and 2 of SYTL4 protein and the C-terminal domain (Figure 1 and Figure 2A). It appears to play a role in the localization of RAB27A to specific sites in a cell [51,52,65]. Upregulation of RAB27A protein in basal forebrain neurons has been associated with mild cognitive impairment and Alzheimer’s disease [52].

Further, specific evolutionarily conserved RAB-binding GTPases function as regulators of membrane trafficking and binding and act as binary molecular switches turned on by binding GTP and off by hydrolyzing GTP to GDP [66]. Their dysfunction through mutations has also been shown to play a crucial role in causing diverse patho-physiologies including X-linked mental retardation associated with autism, epilepsy and macrocephaly. This suggests a major role for specific RAB- binding effector proteins, such as SYTL4 and interacting RAB-activating GTPases (RAB GTPases) in the maintenance of normal neuronal function [51,59,60,61,64,65,66].

#### 3.1.12. Significance of Defect in RAB- Protein Binding Region of N-Terminal Half of SYTL4 Protein due to R (279) C Amino Acid Variant

The potentially deleterious R (279) C amino acid change could critically affect the RAB-binding domain of SYTL4 proteins. Our analysis of the mutant and native SYTL4 protein structure models shows that in the native protein, arginine (R279) forms an apparent salt bridge with aspartic acid (D60). This arginine is part of a large extended loop conformation that appears to be stabilized by the apparent salt bridge formed between it and Asp60 within the RAB-Binding Domain [aa 4-122] and the Ring Domain [aa 43-105].

In our R279C mutant model, cysteine (C279) is located amidst a beta sheet with no apparent hydrogen bonding contacts observed within distance constraints for such interactions. Our modeling thus suggests a potential structural change induced by the R279C variation which eliminates the stabilizing Arg279-Asp60 salt bridge and leads to significant structural changes in the N-terminal half of SYTL4 (Figure 3B). This could very well affect the functionality of the RAB-Binding Domain, not only for the canonical-full-length SYTL4 protein (isoform-1), but also for the truncated SYTL4 protein: isoform-2.

#### 3.1.13. SYTL4-Protein-Protein Interactions with RAB27A and with Other RAB-Family of Genes

As seen in Figure 4, the SYTL4 protein directly interacts with several members of the RAB (Ras-Associated proteins in Brain) family of proteins, RAB3A, RAB8A, RAB27A, and RAB27B. Further, RAB- binding domain of the SYTL4 protein serves as a preferred effector binding site for the GTP-bound form of RAB27A protein that regulates the exocytosis of secretary granules [62,63,64]. Among the RAB- family of genes, *RAB2A*, *RAB11FIP5*, *RAB19*, *RAB39B*, and *RAB43* are known ASD genes [59,60,61] (https://gene.sfari.org/database/human-gene/).

#### 3.1.14. Upregulation of RAB27A Protein Associated with Mild Cognitive Impairment and Alzheimer Disease

RAB27A binds to the N-terminus SLP homology domains 1 and 2 of the SYTL4 protein, and the C-terminal domain seems to play a role in the localization of RAB27A to specific sites in a cell [51,52,65]. It important to note that upregulation of the RAB27A protein in basal forebrain neurons has been associated with mild cognitive impairment and Alzheimer disease [52].

#### 3.1.15. Dysfunction of Conserved RAB-Binding GTPases Play a Role in X-Linked Mental Retardation with Autism

As indicated earlier, specific evolutionarily conserved RAB-binding GTPases do function as regulators of membrane trafficking and binding by acting as binary molecular switches that are turned on by binding GTP and off by hydrolyzing GTP to GDP. Their dysfunction through mutations has been shown to play a crucial role in causing diverse patho-physiologies including X-linked mental retardation associated with autism, epilepsy, and macrocephaly, suggesting a major role for specific RAB-binding effector proteins, such as the SYTL4, and interacting RAB-activating GTPases (RAB- GTPases) in the maintenance of normal neuronal function [51,52,59,60,61,66,67].

#### 3.1.16. *SYTL4* Gene Is Relevant to Neuronal System Function and Disorders

The *SYTL4* gene is relevant for neuronal system development, function and behavior, and is implicated in neurological and psychological diseases (Entrez Gene ID # 94121 (Human); ID # 27359 (Mouse). The *SYTL4* gene expression is down regulated in the dorsal raphe nucleus of patients with major depressive disorders [11]. In addition, in a mouse model of anxiety, significant changes in *Sytl4* were observed among the altered protein networks in the brain proteome [12].

#### 3.1.17. SYTL4 Protein Is Abundantly Expressed in the Bed Nucleus of Stria Terminalis and Is Upregulated in Male Brain

The bed nucleus of stria terminalis (BNST) is a heterogeneous complex limbic forebrain structure, which plays an important role in controlling autonomic, neuroendocrine, and behavioral responses, and is thought to serve as a key relay connecting limbic forebrain structures to hypothalamic and brainstem regions associated with autonomic and neuroendocrine functions [10,14]. Its control of physiological and behavioral activity is mediated by local action of numerous neurotransmitters [68]. Therefore, one could argue that the abundantly-expressed SYTL4 mutant protein in the bed nucleus of stria terminalis could potentially affect normal behavioral responses resulting in an autistic phenotype, given that *Sytl4* is upregulated in the brain of male mice, specifically in the posteromedial area of the medial BNST [9].

Furthermore, in mouse brain, the expression of Sytl4 protein is sexually dimorphic due to the sex hormone (estrogen and testosterone)-specific control of this gene and the developmental influence of sex hormone can lead to enduring effects on brain and behavior [9,69]. Mice with targeted disruptions of the *Sytl4* gene exhibit specific deficits in sex-specific behavior and deficits. *Sytl4* is required for patterning male sexual behavior [9]. Given the increased risk estimates of ASD among males [70,71], the overall odds-ratio for ASD increases if the rare hemizygous mutation on the X-chromosome pertains to gene expression that is known to express in the brain [41].

#### 3.1.18. Targeted Knockout Mutant Mammalian Phenotypes for *Sytl4* Includes Abnormal Behavior and Abnormal Neurological Phenotype

The amino acid changes of SYTL4 R279C in exon 9 in our patient have been determined to severely affect the RAB protein-binding region at its N-terminal [51,52], which may be analogous to the partial or full effect of targeted Null/Knockout mutant *Sytl4* mammalian phenotypes. Gomi et al. [16] found that a targeted (Null/Knockout) mutation for Granuphilin (*Sytl4*) in XY-male mouse with 129P2/OlaHsd- genetic background replaced exons 3–5: targeted mutation 1, Tetsuro Izumi (tm1Tiz): Grn−/Y (*Sytl4* tm1Tiz/Y) particularly affected its endocrine and nervous systems with abnormal corticotroph morphology and hypersecretion of adrenocorticotropin [16,72]. Importantly, the same mammalian-targeted *Sytl4* knock-outs in 129P2/OlaHsd*C3H/He (*Sytl4*tm1Tiz/Sytl4tm1Tiz) and in 129P2/OlaHsd (*Sytl4*tm1Tiz/Y) have been associated with abnormal behavior and neurological phenotypes encompassing alertness, behavioral response to light, circadian rhythm, cognition, consumption behavior, emotion/affect behavior, grooming behavior, impulsive behavior, motor capabilities/coordination/movement, sensory capabilities/reflexes/nociception, sheltering behavior, sleep behavior, vocalization, and social interaction [13,14].

#### 3.1.19. Targeted *Sytl4* Knock Out Mouse Model Studies Affirm That Defective SYTL4 Protein Function is Likely to Effectuate Neurological and Phenotypic Defects

A wide range of functions of the SYTL4 protein is noted for various cellular components, such as intracellular protein transport and positive regulation of protein secretion involving nucleoplasm, cytoplasm, endosome, centrosome, and plasma membrane which govern diverse molecular functions. These include protein and phospholipid binding, zinc and metal ion binding, clathrin binding, and neurexin protein binding. One could postulate that the observed low birth weight and height, as well as the down-slanting palpebral fissures, mild hypertelorism, thin upper lip, and pointed chin seen in our patient could relate to the abnormal SYTL4 protein. These phenotypic abnormalities are perhaps not observable in the Tetsuro Izumi (tm1Tiz) and Grn−/Y (*Sytl4* tm1Tiz/Y) mice models [16,72], however, decreased body weight has been reported in targeted *Sytl4* knock-out (Sytl4tm1Tiz/Sytl4tm1Tiz) mice with 129P2/OlaHsd*C3H/He genetic background. Thus, targeted *Sytl4* knock out mouse model studies affirm that defective SYTL4 protein function may effectuate neurological and phenotypic defects.

#### 3.1.20. SYTL4 Protein Directly Interacts with Proteins Known to Cause Autism

Our protein–protein interactions study of the *SYTL4* gene shows that the SYTL4 protein directly interacts with three other proteins which are known to cause autism, namely, STX1A, SNAP25 and STXBP1 (Figure 4; https://gene.sfari.org/database/human-gene/). In addition, the SYTL4 protein directly interacts with FGF4, STX1B, SNAP29, SNAP23, SNAP47, RAB3A, RAB27A, RAB27B, RAB8A, STX4, STX19, STX3, STX11, and STX2 whose numerous transcriptome-wide isoforms are known as autism genes (https://gene.sfari.org/database/human-gene/).

#### 3.1.21. *SYTL4* Gene Sequence Shows Similarity to a Known Autism Gene: *SYT1*

Moreover, the *SYTL4* gene sequence alignment shows similarity to the *SYT1* gene which is a known autism gene (https://gene.sfari.org/database/human-gene/). Yet another larger Synaptotagmin gene family member, *SYT17*, is also a known ASD gene (https://gene.sfari.org/database/human-gene/).

#### 3.1.22. *SYTL4* Gene Sequence Alignment Shows Similarity to *SYT1*(Synaptotagmin 1) Gene Which is a Known ASD Gene

Although the *SYTL4* gene is presented here as a candidate gene, its transcriptome-wide isoform-level related gene family members, such as *SYT1*, *SYT17,* and *SYT3* are known ASD genes (https://gene.sfari.org/database/human-gene/). Moreover, the *SYTL4* gene sequence alignment shows similarity to the *SYT1* gene [70]. Synaptotagmins are integral membrane proteins of synaptic vesicles thought to serve as Ca (2+) sensors in the process of vesicular trafficking and exocytosis. Calcium binding to synaptotagmin-1 participates in triggering neurotransmitter release at the synapse [70].

#### 3.1.23. Direct Protein-Protein STRING Interactions of the *SYTL4* Gene with Other ASD Genes

More significantly, the SYTL4 protein directly interacts with three known ASD proteins, STX1A, STXBP1 and SNAP25 (Figure 4) (https://gene.sfari.org/database/human-gene/). STX1A (Syntaxin 1A (brain)) encodes a protein involved in the regulation of serotonergic and GABAergic systems and its expression is altered in autism [70]. Rare single gene mutations in *STX1A* have been implicated in ASD. This gene is located at 7q11.23. Common *STX1A* variants are nominally associated with high-functioning autism and Asperger syndrome (https://gene.sfari.org/database/human-gene/). This protein governs the release and uptake of extracellular vesicles in the nervous system and glial cells facilitating transcellular communication [73] and serves as a key molecule in ion channel regulation and synaptic exocytosis (https://gene.sfari.org/database/human-gene/).

The *SYTL4* gene also directly interacts with Syntax binding protein 1 (STXBP1). A frameshift mutation in the *STXBP1* gene has been implicated in a study of quartet families with autism spectrum disorder [74,75,76] and is a known autism gene (https://gene.sfari.org/database/human-gene/). *SYTL4* recruits and binds *STXBP1* to promote exocytosis [26] (http://www.genecards.org/cgi-bin/carddisp.pl?gene=STXBP1&keywords=STXBP]. It is important to note that like *STXBP1*, *SYTL4* is also associated with early onset epileptic encephalopathy and both these genes are expressed in every part of the brain [http://www.genecards.org/). *SYTL4* as well as *STXBP1* are known to be expressed in the frontal cortex and brain and are essential for protein–protein interactions at synapses and the neurotransmitter release cycle in human neurons (http://www.genecards.org/). *STXBP1* is overexpressed in the frontal cortex and even heterozygous mutations cause early onset epileptic encephalopathy, specifically through presynaptic impairment and autism [74,75,76]. Like *STXBP1*, *SYTL4* is also associated with early onset epileptic encephalopathy and is expressed in brain regions (http://www.genecards.org/). Polymorphisms in the *SNAP25* (Synaptosomal-associated protein, 25 kDa) gene are associated with ADHD. *SNAP25*+/− mice also exhibit hyperactivity and cognitive and social impairment [77]. In addition to the SYTL4 protein’s direct interactions with three known ASD proteins, STX1A, STXBP1 and SNAP25, it interacts with 14 other genes, namely, *FGF4, STX1B, SNAP29*, *RAB3A*, *SNAP23*, *RAB27B*, *RAB8A*, *SNAP47*, *STX4*, *STX19*, *STX3*, *STX11*, *STX2*, and *RAB27A*, whose numerous transcriptome-wide isoform-level multiple gene family members are known ASD genes (https://gene.sfari.org/database/human-gene/).

#### 3.1.24. *SYTL4*- Molecular Pathways, Biological Processes and Molecular Functions

Significant *SYTL4* gene-involved molecular pathways are the synaptic vesicle cycle, insulin secretion, AMPK-signaling pathway, and SNARE interactions (Table 1). The synaptic vesicle cycle pertains to synaptic vesicles that are filled with neurotransmitters by active transport, and the diseases that are associated with defects in this cycle are early infantile epileptic encephalopathy; centronuclear myopathy; episodic ataxias; and familial or sporadic hemiplegic migraine. Insulin secretion is regulated by several hormones and neurotransmitters, and the diseases associated with this pathway are defects in the degradation of ganglioside and type II diabetes mellitus. AMPK signaling acts as a sensor of cellular energy status while SNARE interactions mediate the docking of synaptic vesicles with the presynaptic membrane in neurons. Diseases associated with defective SNARE interactions are pseudohypoparathyroidism and cerebral dysgenesis, neuropathy, ichthyosis, and palmoplantar keratoderma or CEDNIK syndrome (Table 1).

The *SYTL4* gene’s extensive biological processes (Table 2) pertain to RAB-protein signal transduction along with synaptic vesicle functions, neurotransmitter secretion, regulation of signaling, glutamate secretion, neuro-muscular synaptic transmission, and axonogenesis, which are all relevant for proper neuronal function, and thus also relevant to autism. The *SYTL4* gene’s molecular functions (Table 3), biological processes (Table 2) and molecular pathways (Table 1) are indicative of its significant role in neuronal function that is meaningful in the causation of high-functioning autism in our proband. Moreover, in recent studies, disturbed *SYTL4* gene function has been associated with neuropsychiatric disorders, such as autism, schizophrenia and depression as well as the immune system. It is also considered a major modulator of central nervous system function [11,12].

#### 3.1.25. Synaptic Dysfunction in Neurodevelopmental Disorders Is Associated with Autism and Intellectual Disabilities

The SYTL4 modulates exocytosis of dense-core granules and secretion of hormones in the pancreas and the pituitary. It interacts with vesicles containing negatively charged phospholipids in a Ca (2+)-independent manner (http://www.genecards.org/). The significant SYTL4 molecular pathways are the synaptic vesicle cycle, insulin secretion, AMPK-signaling pathway, and SNARE interactions (Table 1). Like *SYTL4*, *SYT1*, *STX1A*, *STXBP1* and *SNAP25* genes play an important role in the extravesicular synaptic function of neuronal systems and neurotransmission (https://gene.sfari.org/database/human-gene/; www.genecards.org/). Thus, these proteins are generally involved in the functioning of the synaptic vesicles’ (or neurotransmitter vesicles) cycle to facilitate synaptic vesicle exocytosis by which a synaptic vesicle fuses with the plasma membrane of the pre-synaptic axon terminal and releases its contents in the synaptic cleft, which is essential for propagating nerve impulses between neurons and are constantly created by the cells to facilitate transcellular communication [73].

It should be noted that the *SYTL4* mRNAs are found in extracellular vesicles and stimulate target cells to translate into active protein [78]. The *SNAP25* gene encodes t-SNARE which is involved in the molecular regulation of neurotransmitter release that associates with proteins in vesicle docking and membrane fusion. It may play an important role in the synaptic function of specific neuronal systems [77] (https://gene.sfari.org/database/human-gene/). Similarly, STXBP1 protein is essential for neurotransmission and binds to syntaxin, a component of the synaptic vesicle fusion machinery (https://www.uniprot.org/uniprot/P61764).

Genes that regulate presynaptic processes ultimately affect neurotransmitter release when disturbed [79,80]. Synaptic dysfunction in neurodevelopmental disorders is associated with autism and intellectual disabilities [81,82,83,84] as noted by Baker et al. [80] who reported the first case of a rare missense variant (*I368T*) in the Synaptotagmin1 (*SYT1*) gene causing a human neurodevelopmental disorder. It has a dominant negative effect involving both synaptic vesicle exocytosis and endocytosis [80].

Significant molecular pathways, functions and biological processes (Table 1, Table 2 and Table 3) of the *SYTL4* gene include synaptic vesicle cycle and fusion, exocytosis and neurotransmitter secretion, akin to the molecular functions of its directly interacting known ASD genes: *STXBP1* and *SNAP25*. Therefore, deficiency in the functioning of the SYTL4 protein due to the R (279) C amino acid change affects the canonical structures of the *SYTL4* gene as well as its shorter isoform affecting synaptic vesicle cycle and fusion, exocytosis, and neurotransmitter secretion likely to cause autism and intellectual disabilities.

#### 3.1.26. ASD-Predictive MicroRNAs among Mouse *Sytl4*- Interacting MicroRNAs

Our analysis of 298 validated/predicted microRNA interactions with mouse *Sytl4* gene [34,85,86,87,88] has identified three of five autism-associated serum miRs (60%), namely, miR181b-5p, miR320a, and miR130a-3p, which have good predictive power in serum [34]. Among these three miRs, miR320 has the greatest ASD predictive power reported in serum as well as in the superior temporal gyrus and cerebellar cortex of ASD individuals [34,35,37]. In addition, 5 of 13 (38%) miRs that were differentially expressed in ASD serum samples were predicted to be interacting with mouse *Sytl4* gene [34]. Three of these five miRs, namely, miR130a-3p, miR181b-5p, and miR328 are predicted to be associated with the mouse *Sytl4* gene. They have been shown to be differentially expressed in schizophrenics [31,32].

#### 3.1.27. Dysregulation of miR-320—Most Predictive for ASD in Serum and Brain Tissues

Recently, miR-320, along with miR-197 in human follicular fluid were found to be associated with embryonic development potential [89]. Knocking down miR-320 in mouse oocytes negatively affects embryonic developmental potentially by inhibiting the expression of the Wnt-signaling pathway and therefore miRNAs in human follicular fluid might reflect an effect on embryo quality [89]. Furthermore, autopsy tissue sections showed concordantly dysregulated miR-320a and voltage-dependent anion channel 1 levels in HIV-1 patients suffering from mild cognitive impairment [90]. One could consider dysregulation of miR-320 as the most predictive for ASD in serum and brain tissues, since it was also found to be dysregulated in the superior temporal gyrus of ASD specimens [37]. Additionally, miR-320 and miR-197 are differentially expressed in the ASD cerebellar cortex [35,36]. Given the fact that miR-320 has been predicted to interact with mouse *Sytl4* gene, it gives credence to the *SYTL4* gene as a plausible new gene ASD.

#### 3.1.28. *SYTL4* Interacting miR181b-1- Being Predictive of ASD

The micro RNA second in line to miR320 is miR181b-1 as the most predictive for ASD. It not only showed good predictive power for ASD in serum [34] but is also differentially expressed in the ASD cerebellar cortex [35,36]. Unlike miR-320, miR181b-1 has additionally been found to show altered expression in the cortical regions in schizophrenia [32,33,34], supporting the contention that ASD and schizophrenia share common neurobiological features [32,33,34]. In a recent study, significant down-regulation of miRNA-181b expression in schizophrenics predicted improvement of negative symptoms to treatment, and thus miRNA-181b is predicted to serve as a potential plasma-molecular marker for antipsychotic responses [91].

#### 3.1.29. SYTL4 Interacting miR130a-Being Predictive of ASD

Another potentially important micro RNA is miR130a (miR130a-3p) for prediction of ASD but is lacking corroboration from any ASD brain tissue studies (unlike miR320 and miR181b-1, above). However, like miRNA-181b, miR130a also showed altered expression (serum/cortical) in schizophrenia, again supporting the contention that ASD and schizophrenia share common neurobiological features [32,33,34].

#### 3.1.30. *SYTL4*-Interacting miR106b and miR328 Dysregulated in ASD Cerebellar Cortex and Altered among Schizophrenics

Other SYTL4-associated miRs, miR106b (106b-3p) and miR328, have shown up and down regulation, respectively, in ASD serum and are differentially expressed in the ASD cerebellar cortex [34,35]. In addition, both miRs have also been shown to be altered among schizophrenics [32,33,34].

#### 3.1.31. *SYTL* Interacting miR63, miR103, 5nd miR132 Are Dysregulated in Superior Temporal Gyrus of ASD

Other predicted *SYTL4*-interacting miRs, such as, miR93, miR103 (miR103-1 and miR103-2), and miR132, have been found to be dysregulated in superior temporal gyrus of ASD [37]. Thus, a total of eight microRNAs which are predicted to be associated with mouse *Sytl4* are found to be altered in ASD serum and/or brain, thereby, augmenting our contention that the *SYTL4* gene is a plausible new ASD candidate gene. Fifty percent (4/8) of the ASD-associated miRs (miRs106, miRs130a, miRs181b, and miRs328) that are predicated to interact with the mouse *Sytl4* gene are also known to be associated with schizophrenia, supporting the contention that ASD and schizophrenia share common neurobiological features [34,92,93] (Table 4).

Fifty percent (4/8) of the ASD-associated mirRs (mirRs93, mirRs103, mirRs132, mirRs320) that are predicted to interact with mouse Sytl4 have also been determined to be dysregulated in superior temporal gyrus of ASD [37]. Yet again, 50% (4/8) of the ASD-associated mirRs (mirRs106b, mirRs181b-5p, mirRs320, mirRs328) predicted to interact with the mouse *Sytl4* have also been determined to be differentially expressed in the ASD cerebellar cortex [35]. Over expression of miR142-5p, miR142-3p, miR451a, miR144-3p, and miR21-5p has been reported in ASD brain tissue along with hypomethylation of the promoter region of the miR142 gene in the same samples, suggesting dysregulation of these microRNAs [94]. However, the mouse *Sytl4* gene has not been predicted to interact with any of the miRs that were found to be over expressed by Mor et al. [94]. Furthermore, these five miRs are not represented among the 13 differentially expressed miRs in ASD serum [34] (Table 4). Other studies have also not reported any of the five miRs to be over expressed by Mor et al., [94] based on specific regions of ASD brains, such as the superior temporal gyrus [37] and cerebellar cortex [35].

### 3.2. Transmembrane Protein 187 (TMEM187) Gene

*TMEM187* is an emerging candidate gene for autism with a discussion undertaken on protein interactome networks, expression profiling and microRNA interaction studies.

#### 3.2.1. *TMEM187* Gene Belongs to a Group of Genes Which Host MicroRNA Genes in Their Introns or Exons

At the outset, it should be noted that unlike the *SYTL4* gene, not much is known about the *TMEM187* gene, and there is limited information available regarding its biological processes, molecular pathways and functions or microRNA interactions. The *TMEM187* gene, like *SYTL4*, is a protein-coding gene, but belongs to a group of genes which host microRNA genes in their introns or exons [17]. However, we introduce the *TMEM187* gene as an emerging candidate gene for autism with our mutation analysis of its novel missense variant c.708G>T; p. Gln236His, its STRING-protein interactome network and its expression profiling.

#### 3.2.2. Novel *TMEM187* Missense Variant c.708G>T: Glutamine(Q)236 Histidine(H)

Unlike the *SYTL4* gene variant, *TMEM187* Glutamine(Q)236 Histidine(H) variant in our patient is not found in the Exome Aggregation Consortium (ExAC) database. It is also not found in the listed 261 previous variants currently listed at ensembl.org.

#### 3.2.3. Deleterious and Damaging Nature of the Novel *TMEM187*- Variant

Our extensive analyses of this novel variant, as detailed in the Results section, was often determined to be deleterious or damaging. Glutamine, which is a polar amino acid, was changed to histidine and this alters the protein [43,44].

#### 3.2.4. TMEM187 Protein Is Expressed in Brain

Like *SYTL4,* the *TMEM187* gene is ubiquitously expressed in all systems including all parts of the brain (www.uniprot.org/uniprot/Q14656; www.genecards.org/).

#### 3.2.5. STRING–Gene Interaction Network Study Reveals Direct Protein–Protein Interactions of the TMEM187 Gene with Several Other Known Autism Genes

Although the novel X-linked *TMEM187* missense gene variant c.708G>T; p.Gln236His found in our high-functioning autism patient is not known as an ASD gene, but analysis of the latest STRING network interactions reveal direct interactions with four known ASD genes, namely *HCFC1*, *TMLHE*, *MECP2*, and *GPHN* (https://gene.sfari.org/database/human-gene/) (Figure 7).

#### 3.2.6. Significance of *TMEM187* Protein-Protein Interacting Autism Genes

*HCFC1* is a syndromic ASD gene that interacts with *TMEM187*, while *TMLHE, MECP2* and *GPHN* are rare single gene autism genes (https://gene.sfari.org/database/human-gene/). *HCFC1* is involved in control of the cell cycle with mutations in this X-linked (Xq28) gene associated with intellectual disability [26]. The two genes (*TMEM187* and *HCFC1*) lie just 2kb apart [95]. Over expression of *HCFC1* due to a variant is linked to intellectual disability [26]. A rare mutation in *TMLHE* has been identified with autism (https://gene.sfari.org/database/human-gene/).

The *TMEM187* gene is in linkage with the *MECP2* gene which is a well-known determinant of brain structure, and amino acid variations in the MECP2 protein cause micro-encephalopathy and are also associated with several neurodevelopmental disorders that affect both brain morphology and cognition [96]. Mutations in this gene underlie Rett syndrome, a well-known autism disorder (https://gene.sfari.org/database/human-gene/). Rare single gene mutations in the *GPHN* gene are associated with ASD and this gene encodes a neuronal assembly protein that anchors inhibitors of neurotransmitter receptors to postsynaptic cytoskeleton (https://gene.sfari.org/database/human-gene/).

#### 3.2.7. Significance of Other Protein-Protein Interactions of TMEM187

The TMEM187 protein interacts directly with *UBL4A, RBM25,* and *AKAP4* (Figure 7). Though these genes are not known ASD genes, their other transcriptome-wide isoform-level family member genes are known ASD genes: *UBL7*, *RBM27*, *RBM8A*, *RBMS3* and *AKAP9* (https://gene.sfari.org/database/human-gene/).

Additionally, the TMEM187 protein directly interacts with the LAGE3 (L Antigen Family Member 3) protein (Figure 7), however, *LAGE3* is not a known ASD gene. It is associated with Galloway-Mowat syndrome 2, an X-linked early-onset nephrotic syndrome associated with microcephaly, central nervous system abnormalities, developmental delay, and a propensity for seizures. Brain anomalies include gyration defects such as lissencephaly, pachygyria, polymicrogyria, and cerebellar hypoplasia. Most patients show facial dysmorphism characterized by a small, narrow forehead, large/floppy ears, deep-set eyes, hypertelorism, and micrognathia (www.uniprot.org/uniprot/Q14657).

#### 3.2.8. Other TMEM Proteins Gene Family Members Are Known Autism, Bipolar and Panic Disorder Genes

Other transmembrane proteins gene family members, such as *TMEM231*, are known syndromic autism genes (https://gene.sfari.org/database/human-gene/). This gene is associated with two neurological syndromes: Joubert syndrome-20 [MIM:614970] and Meckel syndrome 11 (https://gene.sfari.org/database/human-gene/; MIM:615397). Additionally, two other members (TMEM132E and TMEM132D) are known to be associated with bipolar and panic disorders [97,98].

#### 3.2.9. X-chromosome Harbors Disproportionately Higher Number of *TMEM187*-Interacting Autism and Nervous System Disorder Genes: Implications for Boys vs Girls Ratio

Except for *GPHN* gene located on chromosome 14, all the other genes (*TMEM187, HCFC1, TMLHE, MECP2, LAGE3* and *SYTL4*) are located exclusively on the long arm of the X-chromosome. The *LAGE3* gene, whose family of genes are clustered together at Xq28, is like that of *TMEM187, HCFC1, TMLHE,* and *MECP2*. SYTL4 is also located on the X-chromosome at q22.1, proximal to the centromere. This augments the assertion that X-chromosome harbors a disproportionately higher number of ASD and nervous system disorder genes [99], and consequently, disproportionately affects more boys than girls, given that the overall odds-ratio for ASD is increased if the rare hemizygous mutation is on the X-chromosome and X-linked genes expressed in the brain [41] as is the case in all six of these X-linked genes (www.genecards.org).

## 4. Materials and Methods

### 4.1. Clinical Report

The 7-year-old male proband was the only child born to healthy young non-consanguineous parents. There was no family history of genetic disorders, malformations, epilepsy, autism, or intellectual disability. Our proband was the product of a 38-week pregnancy to a primigravida mother via an uncomplicated C-section due to a transverse presentation. The Apgar scores were 9 and 9 at 1 and 5 min, respectively. The birth weight was 3600 gm (55th percentile), length was 52 cm (85th percentile), and head circumference was 35 cm (55th percentile).

The proband was evaluated in the Department of Pediatric Neurology, Hospital Universitario Quirón, Madrid, Spain at the age of 4.5 years due to longstanding impairment in social and communicative functioning. Although an early intervention program was established in the first months of life for motor, cognitive, speech development, and social behavior, the proband exhibited mild psychomotor delay during his first years of life. He walked unsupported at 11 months but had significant problems with walking, squatting or dressing at 4.5 years of age. First bi-syllabic babbling occurred at 18 months; at the age of 3 years, he only spoke words without making sentences. His social development was markedly affected; he showed atypical behaviors, refused playing with other children, had a limited amount of interests, and eye contact was minimal.

His weight was 19 kg (65th percentile) and height was 109 cm (65th percentile). He had down-slanting palpebral fissures, mild hypertelorism, thin upper lip, and a pointed chin. Conventional genetic studies (karyotype and array comparative genomic hybridization) showed no abnormalities. The neurological exam was normal. He had impaired social interaction during the examination and lacked eye contact, had peculiar language (echolalias, verbosity, and abnormal pitch), stereotyped mannerisms, and restricted patterns of interest. Brain MRI and sleep video-EEG tests displayed normal results. Cognitive assessment using the Wechsler Preschool and Primary Test of Intelligence-III (WPPSI-III) revealed a verbal and non-verbal IQ at above-average level without significant discrepancies. The Behavior Assessment System for Children (BASC) completed by his parents and preschool teachers revealed significant problems in “social skills,” “adaptability” and “atypicality” domains.

At the age of 6 years, his neurological examination remained normal, but he had an unusually high-pitched voice with stereotyped phrases and echolalia. He tended to perseverate on repetitive interests and activities (chronology of history, borders of countries). His eye contact was inconsistent and poorly integrated with other communicative efforts. He reacted aversely to sensory stimuli (e.g., loud noise, flavors). Autism Spectrum Screening Questionnaire (ASSQ), the Autism Diagnostic Interview-Revised (ADI-R), and the Autism Diagnostic Observation Scale (ADOS, Module 3) were administered. His total score on the ASSQ was 31 and 29 according to the evaluation by the teachers and parents (high-functioning autism cut-off = 22 and 19, respectively). His ADI-R algorithm scores were 15 on the social domain (autism cut-off = 10), 11 on the communication domain for verbal children (autism cut-off = 8), and 5 on the repetitive behavior’s domain (autism cut-off = 3). His total score on the ADOS communication and social algorithm items were 6 and 9 (autism cut-off = 3 and 6, respectively). The clinical and neuropsychological evaluations were consistent with high-functioning autism. The patients allowed for us to undertake research investigations. The study was approved by the local ethics committees on January 7, 2016 and was conducted in accordance with the ethical principles of the Declaration of Helsinki and Good Clinical Practice standards. Informed consent was obtained from parents, with the child giving assent.

### 4.2. Genomic Investigations

Exome sequencing was performed using genomic DNA isolated (MagnaPure, Roche Applied Science, Manheim, Germany) from whole blood from the proband and parents. Libraries were prepared using the Ion AmpliSeq™ Exome Kit (Life Technologies, Carlsbad, California, USA) and quantified by qPCR. The enriched libraries were prepared using Ion Chef™ and sequenced on PI™ Chip in the Ion Proton™ System (Life Technologies) to provide >90% of amplicons covered with at least 20X. Signal processing, base calling, alignment and variant calling were performed on a Proton™ Torrent Server using the Torrent Suite™ Software (v4.4 Life Technologies, Carlsbad, CA, USA). Variants were annotated using Ion Reporter™ Software with the human genome reference assemble GRCh37 (hg19) and pedigree analysis performed using the Genetic Disease Screen (GDS) trio workflow.

Candidate variants were visualized using IGV (Integrative Genomics Viewer, Cambridge, MA, USA) and evaluated based on stringent assessments at both the gene and variant levels, taking into consideration both the patient’s phenotype and the inheritance pattern. Variants in the *SYTL4* and *TMEM187* genes were recognized as probable pathogenic and confirmed by Sanger sequencing. However, due to the lack of cooperation from other family members, additional testing was not available.

### 4.3. Modeling of Native and R279C Variant for SYTL4 Gene

The theoretical atomic models of native and R279C *SYTL4* were constructed using I-TASSER [20,100,101]. With human SYTL4 sequence (UniProtKB Accession # = Q96C24) as query, multiple sequence-template alignments were initially generated by the meta-threading program LOMETS [20,102,103], followed by generation of the predicted atomic structures. Native SYTL4 matched well with several moderately high-scoring templates corresponding to synaptotagmin family members, with an estimated TM score of 0.5 ± 0.15 and RMSD of 12.2 ± 4.4 Å. The R279C variant also matched to synaptotagmin family member templates, with slightly lower scores, yielding a TM score and RMSD of 0.45 ± 0.15 and 13.5 ± 4.0 Å, respectively.

Both structures were close to the threshold (TM score >0.5) for correct topology. Molecular graphics and analyses were performed with the UCSF Chimera package. Chimera is developed by the Resource for Biocomputing, Visualization, and Informatics at the University of California, San Francisco (supported by NIGMS P41-GM103311) [85].

### 4.4. MicroRNAs

MicroRNAs (miRNAs / miRs) play a key role in the transcriptional networks of the developing human brain, as regulators of gene expression. Autism spectrum disorder (ASD), being a complex neurodevelopmental disorder, is characterized by multiple deficits in communication, social interaction and behavior [34]. Vasu et al. [34] examined the serum expression profiles of 125 neurologically relevant miRNAs expression profiles in 55 individuals with ASD. These neurologically relevant miRNAs represented pathways involved in axon guidance, TGF-beta signaling, MAPK signaling, adherents’ junction, regulation of actin cytoskeleton, oxidative phosphorylation, hedgehog signaling, focal adhesion, mTOR signaling, and Wnt signaling [34].

Vasu et al. [34] found that only 13 miRNAs (out of the selected 125 miRs; ~10%) were differentially expressed among the 55 ASD individuals compared to the controls in their serum. Of these 13, miR151a-3p, miR181b-5p, miR320a, miR328, miR433, miR489, miR572, and miR663a were downregulated (61.5%), while miR101-3p, miR106b-5p, miR130a-3p, miR195-5p, and miR19b-3p were upregulated (38.5%) [34]. Furthermore, of these 13 miRs, only five miRs (38.5%), namely, miR181b-5p, miR320a, miR572, miR130a-3p and miR19b-3p had high values for sensitivity, specificity and area under the curve, thereby showing good predictive power for distinguishing individuals with ASD [34]. Therefore, it was decided to screen for the presence of these five miRNAs with good ASD predictive power, namely, miR181b-5p, miR320a, miR572, miR130a-3p and miR19b-3p as well as the other five miRs that were also differentially expressed among the 55 ASD individuals among the 298 validated/predicted microRNA interactions of mouse *Sytl4* gene [86,87,88,104]. Additionally, we screened for the presence of other significantly ASD-associated miRs from brain-specific micro RNA expression studies by others [35,36,37].

## 5. Conclusions

1. *TMEM187* as well as *SYTL4* genes are: X-linked and both located on the long arm of the X-chromosome at Xq28 and Xq22.1, respectively;

2. Both *Q236H TMEM187* and R279C SYTL4 gene variants have been predicted to be damaging or deleterious by SIFT, PolyPhen2, MutationTaster, Provean, and LRT variant calling programs;

3. Both *TMEM187* and *SYTL4* mRNAs are found in extracellular vesicles and stimulate target cells to translate into active protein [17], and the release and uptake of extracellular vesicles in the nervous system and glial cells provides novel mechanisms of transcellular communication [73];

4. Together, *TMEM187* and *SYTL4* genes directly interact with seven known ASD genes: four and three ASD genes, respectively (Figure 4 and Figure 7; https://gene.sfari.org/database/human-gene/);

5. Another transmembrane protein gene family member, *TMEM231*, is a known syndromic autism gene: *TMEM231* gene is associated with two neurological syndromes: Joubert syndrome-20 [MIM:614970] and Meckel syndrome 11 (https://gene.sfari.org/database/human-gene/; MIM:615397);

6. The SYTl4-RAB-binding protein RAB27A is specifically associated with mild cognitive impairment and Alzheimer disease [52];

7. RAB-binding GTPases play a crucial role in causing diverse patho-physiologies including X- linked mental retardation (intellectual disability) associated with autism, epilepsy, and macrocephaly, suggesting a major role for specific RAB-binding effector proteins, such as SYTL4, and interacting RAB-activating GTPases (RAB GTPases) in the maintenance of normal neuronal function [51,59,60,61,64,65,66];

8. Two other members of the transmembrane protein gene family, *TMEM132E* and *TMEM132D,* are known to be associated with bipolar and panic disorders, respectively [97,98];

9. One of the *TMEM187* genes’ related phenotypes is schizophrenia (GWAS catalog for TMEM187 gene: Gene relation via enhancers containing phenotype SNP: Enhancer ID: GH0XJ153980; https://genecards.weizmann.ac.il/geneloc-bin/display_map.pl?chr_nr=0X&range_type=gh_id&gh_id=GH0XJ153941#GH0XJ153941). Similarly, our extensive ASD predictive mouse Sytl4-interacting microRNAs study reveals that 50% of the ASD-associated miRs are known to be associated with schizophrenia (Table 4);

10. A recent large study of gene expression patterns from postmortem brain tissues found transcriptome-wide isoform-level dysregulation in ASD, schizophrenia, bipolar disorder, panic disorder, and other related neurological disorders [105], supporting our above findings in *SYTL4* and *TMEM187* gene variants that ASD and schizophrenia share common neurobiological features [34,92,93];

*11.* Both *SYTL4* and the *TMM187* genes are ubiquitously expressed, including in the brain [9,10,14] (www.GeneCards.Org; www.Uniprot.Org). The overall odds-ratio for ASD is increased if rare hemizygous mutations on the X-chromosome in male patients are found as these genes have known expression in the brain [41].

Given the above analytical analyses, there is evidence to support that the missense mutations seen in both the *TMEM187* and *SYTL4* genes, either synergistically or individually, are causal mutations for the high-functioning autism seen in our patient. Consequently, both genes are proposed as novel autism candidate genes.

It is probable that both gene variants synergistically are causative of the high-functioning autism seen in our patient. Oligogenic heterozygosity or involvement of more than one gene observed in patients, suggests a new potential mechanism in the pathogenesis of autism spectrum disorders [106], further supporting the suggestion that the multifactorial model of ASD risk or monogenic may be too simplistic even for the most penetrant causes of ASD [107]. Pathogenicity of mutations individually or synergistically would require biological assays, such as in-vivo models, for study. Meanwhile, publications of similar findings by others in the study of autism involving either of these two gene variants would lend credence to our findings and assertions.

## Figures and Tables

**Table 1:** SYTL4 Molecular Pathways and Associated Diseases.

Pathway ID	Pathway Description	Count in Gene Set	False Discovery Rate	Functional Description
4721	Synaptic vesicle cycle	3	0.0161	Communication between neurons is mediated by the release of neurotransmitter from synaptic vesicles (SVs). At the nerve terminal, SVs cycle through repetitive episodes of exocytosis and endocytosis. SVs are filled with neurotransmitters by active transport. DISEASES: Early infantile epileptic encephalopathy; Centronuclear myopathy; Episodic ataxias; Familial or sporadic hemiplegic migraine
4911	Insulin secretion	3	0.0161	Insulin secretion is regulated by several hormones and neurotransmitters. Peptide hormones, such as glucagon-like peptide 1 (GLP-1), increase cAMP levels and thereby potentiate insulin secretion via the combined action of PKA and Epac2. Acetylcholine (Ach), a major parasympathetic neurotransmitter. DISEASES: Type II diabetes mellitus; Defects in the degradation of ganglioside.
4152	AMPK signaling pathway	3	0.0325	AMP-activated protein kinase (AMPK) is a serine threonine kinase that is highly conserved through evolution. AMPK system acts as a sensor of cellular energy status.
4130	SNARE interactions	2	0.0432	SNARE proteins (an acronym derived from “SNAP (Soluble NSF Attachment Protein) Receptor”). The primary role of SNARE proteins is to mediate vesicle fusion, that is, the fusion of vesicles with their target membrane-bound compartments. The best studied SNAREs are those that mediate docking of synaptic vesicles with the presynaptic membrane in neurons. DISEASES: Pseudohypoparathyroidism and Cerebral dysgenesis, neuropathy, ichthyosis, and palmoplantar keratoderma syndrome; CEDNIK syndrome.

**Table 2:** SYTL4- Network: Biological Processes

Pathway ID	Pathway Description	Count in Gene Set	False Discovery Rate
GO:0048489	synaptic vesicle transport	8	1.14 × 10^−9^
GO:0097479	synaptic vesicle localization	8	1.14 × 10^−9^
GO:0016079	synaptic vesicle exocytosis	7	1.79 × 10^−9^
GO:0016082	synaptic vesicle priming	4	1.14 × 10^−7^
GO:0031629	synaptic vesicle fusion to presynaptic membrane	4	5.45 × 10^−7^
GO:0007269	neurotransmitter secretion	6	3.06 × 10^−6^
GO:0048167	regulation of synaptic plasticity	5	0.000171
GO:0032482	RAB protein signal transduction	4	0.000701
GO:0031630	regulation of synaptic vesicle fusion to presynaptic membrane	2	0.00156
GO:0014047	glutamate secretion	3	0.00165
GO:0007268	synaptic transmission	6	0.0115
GO:0023051	regulation of signaling	1	0.0211
GO:0050803	regulation of synapse structure or activity	4	0.0249
GO:0050804	modulation of synaptic transmission	4	0.0356
GO:0007274	neuromuscular synaptic transmission	2	0.0385
GO:0065008	regulation of biological quality	1	0.0389
GO:0007409	axonogenesis	5	0.0393

**Table 3:** Molecular Function (GO)

Pathway ID	Pathway Description	Count in Gene Set	False Discovery Rate	Functional Description and Associated Diseases
GO:0019905; GO:0017075	Syntaxin binding	6	5.46 × 10^−7^	Syntaxin binding is essential for neurotransmission: syntaxin is a component of the synaptic vesicle fusion machinery. Mutations in Syntaxin binding protein 1 (STXBP1) have been associated with infantile-epileptic encephalopathy-4 [28].
GO:0005484	SNAP receptor activity	4	0.000161	SNAPRE activity also regulates neurotransmitter release to ensure vesicle-to-target specificity (SNAP receptors implicated in vesicle targeting and fusion [29].
GO:0019003	GDP binding	3	0.019	The trimeric-G-protein (GTP binding proteins) play a pivotal role in the signal transduction pathways for numerous hormones and neurotransmitters [30].

**Table 4:** Showing the Five Autism Predictive Human Serum MicroRNAs with Predicted Interaction with Mouse *Sytl4* Gene.

Sytl4-miR=ASD-miR	Mouse Sytl4- miRs: Predicted Interactions with ASD & Schizophrenia- Associated miRs	Validation	Reference
miR93	Sytl4	predicted	*MGI:1351606c*
miR93	ASD	Dysregulated in superior temporal gyrus of ASD	Stomova, et al., 2015 [37]
miR103-1; miR103-2	Sytl4	Predicted	*MGI:1351606c*
miR103	ASD	Dysregulated in superior temporal gyrus of ASD	Stomova, et al., 2015 [37]
miR106b	Sytl4	Predicted	*MGI:1351606c*
miR106b-5p (miR106b) *	ASD	Upregulated in ASD-serum; differentially expressed in ASD cerebellar cortex	Vasu, et al., 2014 [34] and Abu-Elneel K, et al., 2008 [35]
miR106b	Schizophrenia	Altered expression (serum/cortical)	Vasu, et al., 2014 [34] and Shi, et al., 2012 [33], Beveridge and Cairns, 2012 [32].
miR130a	Sytl4	Predicted	*MGI:1351606c*
miR130a-3p (miR130a) **	ASD	Good predictive power for ASD- serum	Vasu, et al., 2014 [34]
miR-130a	Schizophrenia	Altered expression (serum/cortical)	Vasu, et al., 2014 [34]; Shi, et al., 2012 [33], Beveridge and Cairns, 2012 [32]
miR132	Sytl4	Predicted	*MGI:1351606c*
miR132	ASD	Dysregulated in superior temporal gyrus of ASD	Stomova, et al., 2015 [37]
miR181b-1 (miR181b) ***	Sytl4	Predicted	*MGI:1351606c*
miR181b-2	Sytl4	Predicted	*MGI:1351606c*
miR181b-5p (miR181b/b1) ***	ASD	Good predictive power for ASD in serum; differentially expressed in ASD cerebellar cortex	Vasu, et al. 2014 [34]. Abu-Elneel K, et al. 2008 [35]. Ghahramani Seno, et al., 2011 [36].
miR181b	Schizophrenia	Altered expression (serum/cortical)	Vasu, et al., 2014 [34]; Shi, et al., 2012 [33], Beveridge and Cairns, 2012 [32].
miR320	Sytl4	Predicted	*MGI:1351606c*
miR320a (miR320) ****	ASD	Good predictive power for ASD in serum	Vasu, et al., 2014 [34]
miR320	ASD	Dysregulated in superior temporal gyrus of ASD	Stomova, et al., 2015 [37]
miR320	ASD	Differentially expressed in ASD cerebellar cortex	Abu-Elneel K, et al. 2008 [35]
miR328	Sytl4	Predicted	*MGI:1351606c*
miR328	ASD	Down regulated in serum; differentially expressed in ASD cerebellar cortex	Vasu, et al., 2014 [34] and Abu-Elneel K, et al., 2008 [35]
miR328	Schizophrenia	Altered expression (serum/cortical)	Vasu, et al., 2014 [34] and Shi, et al., 2012 [33]; Beveridge and Cairns, 2012 [32]

* http://www.mirbase.org/cgi-bin/mirna_entry.pl?acc=MI0000407; ** http://www.mirbase.org/cgi-bin/mirna_entry.pl?acc=MI0000156; *** http://www.mirbase.org/cgi-bin/mature.pl?mature_acc=MIMAT0000673; **** http://www.mirbase.org/cgi-bin/mirna_entry.pl?acc=MI0000542; C. http://www.informatics.jax.org/interaction/explorer?markerIDs=MGI:1351606.
